# Pleuro-Pneumonia

**Published:** 1884-10

**Authors:** 


PLEURO-PNEUMONIA.—The Agricultural Department and the Western Health authorities have done themselves credit by so rapidly diagnosing and informing the public of the presence of pleuro-pneumonia in the West. We trust that they will succeed in impressing Congress and the State Legislatures with the absolute necessity of taking forcible measures with the disease. Every infected herd ought to be quarantined, and diseased animals killed. There is no other way, and this is the cheapest in the end. We trust that no respectable veterinarians in this country will advocate any other policy. It is true that some strong arguments can be brought forward in favor of inoculation. But inoculation has not yet been proved beyond a doubt efficient, while the stamping-out process has. And we cannot afford to experiment in this country.

The present epidemic furnishes an opportunity for American philologists to discover, cultivate, and perhaps modify the micro-organism of pleuro-pneumonia, since such undoubtedly exists. C.

